# Persistence of the Recombinant Genomes of Woodchuck Hepatitis Virus in the Mouse Model

**DOI:** 10.1371/journal.pone.0125658

**Published:** 2015-05-05

**Authors:** Danzhen Pan, Yong Lin, Weimin Wu, Jingjiao Song, Ejuan Zhang, Chunchen Wu, Xinwen Chen, Kanghong Hu, Dongliang Yang, Yang Xu, Mengji Lu

**Affiliations:** 1 Department of Microbiology, School of Basic Medicine, Tongji Medical College, Huazhong University of Science and Technology, Wuhan, China; 2 Wuhan Institute of Virology, Chinese Academy of Sciences, Wuhan, China; 3 Department of Infectious Disease, Union Hospital, Tongji Medical College, Huazhong University of Science and Technology, Wuhan, China; 4 Institute of Virology, University Hospital of Essen, Essen, Germany; 5 Division of Clinical Immunology, Tongji Hospital, Tongji Medical College, Huazhong University of Science and Technology, Wuhan, China; Indiana University, UNITED STATES

## Abstract

Hydrodynamic injection (HI) with a replication competent hepatitis B virus (HBV) genome may lead to transient or prolonged HBV replication in mice. However, the prolonged HBV persistence after HI depends on the specific backbone of the vector carrying HBV genome and the genetic background of the mouse strain. We asked whether a genetically closely related hepadnavirus, woodchuck hepatitis virus (WHV), may maintain the gene expression and replication in the mouse liver after HI. Interestingly, we found that HI of pBS-WHV1.3 containing a 1.3 fold overlength WHV genome in BALB/c mouse led to the long presence of WHV DNA and WHV proteins expression in the mouse liver. Thus, we asked whether WHV genome carrying foreign DNA sequences could maintain the long term gene expression and persistence. For this purpose, the coding region of HBV surface antigen (HBsAg) was inserted into the WHV genome to replace the corresponding region. Three recombinant WHV-HBV genomes were constructed with the replacement with HBsAg a-determinant, major HBsAg, and middle HBsAg. Serum HBsAg, viral DNA, hepatic WHV protein expression, and viral replication intermediates were detected in mice after HI with recombinant genomes. Similarly, the recombinant genomes could persist for a prolonged period of time up to 45 weeks in mice. WHV and recombinant WHV-HBV genomes did not trigger effective antibody and T-cell responses to viral proteins. The ability of recombinant WHV constructs to persist in mice is an interesting aspect for the future investigation and may be explored for *in vivo* gene transfer.

## Introduction

Recently, hepatitis B virus (HBV) mouse models based on the hydrodynamic injection (HI) were proven to be useful to study HBV replication, persistence and clearance, and test certain antiviral therapy strategies, though there is no viral spread in this model [1–10]. Yang *et al*. reported that HI of replication-competent, overlength HBV genome in B10.D2 mice resulted in high levels of HBV replication and replication levels decreased after one week. HBV transcripts and replicative intermediates disappeared from the mouse liver after two weeks, coincident with the appearance of antiviral CD8^+^ T cells. In contrast, HBV persisted for more than 10 weeks after HI in NOD/Scid mice or mice treated with immunosuppressive drugs, clearly showing that HBV clearance depends on the host immune response [3, 8]. Later, Huang *et al*. reported that HI of a replication-competent overlength HBV genome in an AAV vector led to the persistent HBV replication and gene expression in C57BL/6 mice [1]. It was speculated that the unknown sequences in the backbone of pAAV/HBV1.2 regulated the long-term maintenance or expression of HBV genome *in vivo*. And the genetic background of recipient mice, which correlates with the strength of immune responses against viral antigen also determined the outcome after HI. Both the vector backbone and the host genetic background were found to be important for HBV persistence [1]. The HBV mouse models based on HI were explored for different studies, for example, to examine the immunological requirements of HBV clearance or to test HBV vaccination [4]. We also used HI of a 1.3 fold overlength HBV genome in the vector of pBluescript II SK(+), which led to a temporary HBV replication in BALB/c and C57BL/6 mice, to study HBV drug resistant mutants and other genetic variants [2, 10,11].

Woodchuck hepatitis virus (WHV) is a member of the family *Hepadnaviridae* discovered in 1978 [12] and infects the natural host of eastern woodchucks (*Marmota monax*) in Northern America. WHV and HBV genomes show a homology of 60–65% at the level of nucleotide (nt) sequences [13]. The woodchuck model has been proven to be a useful model for studies on hepadnaviral infection and pathogenesis and for the evaluation of antiviral drugs [14,15].

Based on the high genetic similarity of HBV and WHV, we asked whether the overlength WHV genome may be able to replicate or maintain the gene expression in mouse liver after HI in BLAB/c mouse. Surprisingly, we found that WHV genome and WHV proteins could be detected in mice for a prolonged period up to 40 weeks, though at low levels. To examine whether other proteins could be expressed by recombinant WHV genomes, we replaced the coding regions of WHV surface antigen (WHsAg) in WHV genome by the corresponding coding sequences for HBsAg to construct recombinant WHV-HBV genomes. The recombinant WHV-HBV genomes could also persist and express WHV and HBV proteins in mouse liver for a prolonged period of time after HI. Thus, WHV genome may be explored as a vector for long term expression of desired genes in the mouse liver.

## Materials and Methods

### Ethics statement

Fifty mice used in this study were bred in the Laboratory Animal Centre of Tongji Medical College, Huazhong University of Science and Technology under specific pathogen free conditions. The mice were fed diets daily and had access to water by drinking bottle. The experiments were conducted in strict accordance with the Guide for the Care and Use of Laboratory Animals and were approved by the Institutional Animal Care and Use Committee of Tongji Medical College, Huazhong University of Science and Technology (Permit Number: 2011–347). All surgery was performed under sodium pentobarbital anesthesia, and all efforts were made to minimize suffering.

### HI in BALB/c mice

Female BALB/c mice at 6–8 weeks of age were obtained from the Animal Center of Hubei province, China. Ten μg of plasmids were injected into the tail vein of mice in a volume of 0.9% NaCl solution equivalent to 8% of the mouse body weight [4]. The total volume was delivered within 8 seconds (s).

### Detection of the encapsidated viral DNA by PCR

50 μl of mouse sera were digested with 60 units of DNaseI (TaKaRa) at 37°C overnight to eliminate the residual input DNA. Encapsidated viral DNA was then released by proteinase K digestion and purified by viral DNA kit (Omega Bio-Tek) according to the manufacturer’s instructions. Encapsidated viral DNA in mice challenged by pBS-WHV1.3 or pWHV-HBV-Sa was detected by PCR using the primers WQp1 and WQp2 (S1 Table). Meanwhile, encapsidated viral DNA in mice injected by pBS-HBV1.3, pWHV-HBV-SS or pWHV-HBV-MS was detected by PCR using the primers HQp1 and HQp2 (S1 Table). The specificity of PCR products was verified by agarose gel electrophoresis.

### Detection of WHcAg, WHsAg and HBsAg in liver sections by immunohistochemical staining (ICS)

Liver samples were collected from mice sacrificed at the indicated time points, formalin-fixed, and paraffin-embedded. Paraffin sections were stained with rabbit antibodies to WHV core antigen (WHcAg) and WHsAg, and a goat antibody against HBsAg (Thermo Scientific, IL, USA), respectively, following the procedure described previously [16].

### Construction of recombinant WHV-HBV genomes

A 1.3 fold overlength WHV genome (nt 1050–2190, GenBank accession no. J04514) was cloned into pBluescript II SK(+) (Agilent Technologies, CA) at the restriction sites *Kpn*I and *Eag*І, resulting in a new vector pBS-WHV1.3 (kindly provided by Dr. Zhongji Meng). Similarly, pBS-HBV1.3 containing a 1.3 fold overlength HBV genome (nt 1038–1984, GenBank accession no. AY220698) was constructed by insertion at the restriction sites *Pst*I and *Sac*I (kindly provided by Dr. Lei Li).

**Fig 1:**
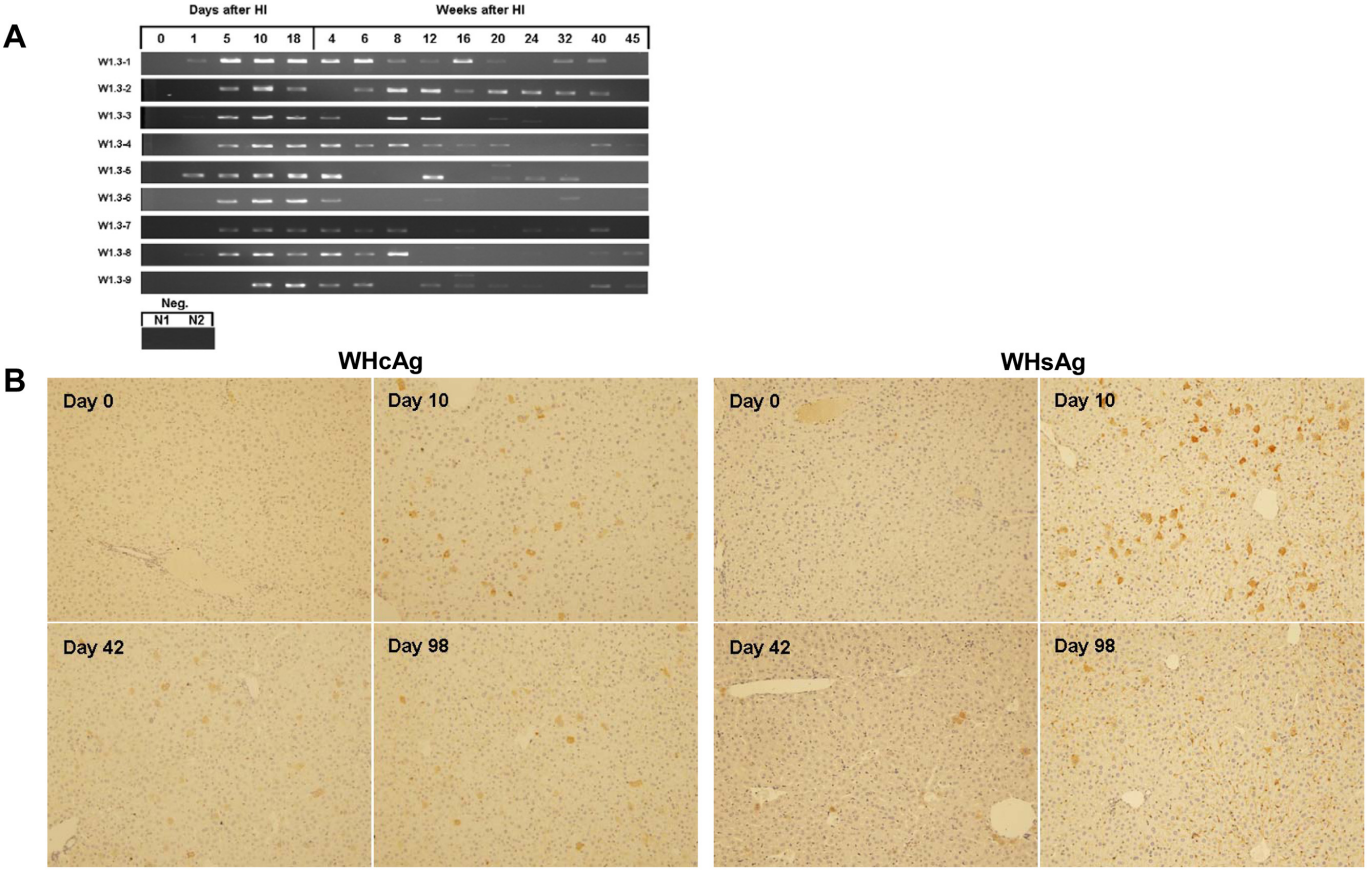
Serum WHV DNA and intrahepatic viral gene expression after HI with pBS-WHV1.3. Nine BLAB/c mice (W1.3–1 to 9) were hydrodynamically injected with pBS-WHV1.3. (A) Mouse sera were collected at the indicated time points after HI. Encapsidated WHV DNA in sera was detected by PCR. Products of the qualitative PCR were visualized by agarose gel electrophoresis. Neg.: sera from two mice (N1, 2) challenged by saline. (B) WHcAg and WHsAg expression in liver were detected by ICS. Liver samples from three mice were taken at days 0, 10, 42, and 98 after HI and stained with rabbit antibodies to WHcAg and WHsAg, respectively. A set of representative samples was shown. Magnification: 200×.

The plasmid pMCS5 has been constructed in our previous work and contains WHV preS2-S region (nt 107–987, GenBank accession no. J04514) with the middle part replaced by HBV sequence (nt 509–605, GenBank accession no. Y07587), encoding a chimeric middle surface antigen with WHV preS2 plus WHsAg amino acid (aa) 1–116, HBsAg aa 121–147, and WHsAg aa 144–222 [17]. The plasmids pBS-WHV1.3 and pMCS5 both were digested with the restriction enzymes *Xba*I and *Apa*I. The fragment nt 382–887 of WHV genome in pBS-WHV1.3 was replaced by the corresponding fragment from pMCS5, resulting in the plasmid pWHV-HBV-Sa. pWHV-HBV-Sa contained 1.3 fold overlength WHV genome, replaced with HBsAg a-determinant (aa 121–147) coding region (Fig 1A).

In the plasmid of pWHV-HBV-SS, the coding region of WHsAg in WHV genome was replaced by that of major HBsAg (Fig 1A). The fragment A with WHV genomic sequence nt 1050–3052 was generated from pBS-WHV1.3 by digestion with the restriction enzymes *Kpn*I and *Pst*I. The fragment Sb1 with nt 3047–298 and fragment C with nt 967–1915 were generated from pBS-WHV1.3 by PCR. Complementary, the fragment Sb2 with nt 157–838 was generated from pBS-HBV1.3 by PCR. The fragment Sb1 was ligated with the fragment Sb2 encoding major HBsAg by fusion PCR, resulting in the fragment SB (S1 Fig). pBS-WHV1.3 was cleaved with two restriction enzymes *Kpn*I and *Nsi*I to produce the vector backbone with a short WHV genomic sequence (nt 1915–2190). Finally, the four fragments (A, SB, C, and the vector backbone) were ligated at the *Kpn*I, *Pst*I, *Hpa*I and *Nsi*I sites in order, generating the plasmid of pWHV-HBV-SS (S1 Fig). Using the same strategy, the coding region of middle HBsAg (nt 3207–837) was inserted into pBS-WHV1.3 and replaced WHV preS2-S region (nt 116–964), generating the plasmid of pWHV-HBV-MS (Fig 1A, S1 Fig). The primers used in PCR were listed in S1 Table. All PCR products were cloned into pMD-19T vector (TaKaRa, Dalian, China) and sequenced by a commercial service (Yingjun Biotechnology Company, Shanghai, China) to verify the correctness of the sequences, before they were used for cloning.

A plasmid with the mutated WHV genome based on pWHV-HBV-Sa, pSaΔP, was constructed by site-directed mutagenesis. In this clone, the start codon ATG of WHV polymerase was mutated to ACG, preventing the expression of WHV polymerase. In detail, two fragments A1 (nt 1050–2438) and A2 (nt 2421–3052) were generated from pWHV-HBV-Sa by PCR using the primers of A1-F/ΔP-R and ΔP-F/A2-R, respectively (S2 Table). The fragments A1 and A2 were ligated by fusion PCR, resulting in the fragment AΔP, which contained the mutated start codon of WHV polymerase, ATG to ACG. After digestion with the restriction enzymes *Kpn*I and *Pst*I, the fragment AΔP was reintroduced into the backbone pWHV-HBV-Sa and replaces the wild type sequence, generating the plasmid pSaΔP.

### Transient transfection and detection of HBsAg expression in hepatoma cells

Different plasmids including recombinant WHV-HBV genomes, pBS-WHV1.3, pBS-HBV1.3 and pHBc were transiently transfected into human hepatoma Huh7 [18] and HepG2 [19] cells. Huh7 and HepG2 cells were directly obtained from China Center for Type Culture Collection, the catalogue number were GDC134 and GDC024, respectively. Huh7 and HepG2 cells were cultured in Dulbecco’s Modified Eagle Medium (DMEM, Gibco, Gaithersburg, MD), supplemented with 10% of fetal calf serum (FCS). Cells were seeded in 6-well plates at approximately 60% confluence. After 24 hours (h) cells were transfected with Lipofectamine 2000 (Invitrogen) according to the manufacturer’s instructions [4]. The supernatants of cell cultures were collected at 24, 48, 72, and 96 h after transfection and subjected to the detection of HBsAg expression by enzyme linked immuno-sorbent assay (ELISA) (Kehua, Shanghai, China). The cut off value was set as 2.1 times over negative controls. To control the transfection efficiency, pBS-WHV1.3 and the recombinant constructs were co-transfected with pEGFP-N1 in Huh7 cells. Under the fluorescence microscope, it was shown that about 30% of the transfected cells expressed GFP. There was no obvious difference in the transfection efficiency of WHV and recombinant WHV-HBV genomes.

### Serological assays

HBsAg and anti-HBs in mouse sera were detected by the respective commercial ELISA kits (Kehua) according to the manufacturer’s instructions. Mouse sera were tested at 1:10 dilution and the results were read at optical density (OD) 450 nm. The cut off value was set as 2.1 times over negative controls.

### Detection of viral replication intermediates by southern blot (SB)

Encapsidated viral DNA in mouse liver samples was analyzed by Southern blotting as described previously [16]. Briefly, the mouse liver tissue was homogenized in 1 ml of ice-cold Tris-EDTA buffer. Nonidet P-40 (final concentration 0.5%) was added, and the samples were incubated on ice for 30 min. Nuclei was pelleted by centrifugation. Supernatants were adjusted to 10 mM MgCl_2_ and treated with 100 μg/ml DNase I (Roche, Mannheim, Germany) at 37°C for 30 min. The reaction was stopped by the addition of EDTA to a final concentration of 25 mM. Next, proteins were digested with 0.5 mg/ml proteinase K (Qiagen, Düsseldorf, Germany) in the presence of 1% sodium dodecyl sulfate at 55°C for 2 h. Encapsidated viral DNA was purified by phenol/chloroform (1:1) extraction followed by isopropanol precipitation by adding 15 μg of tRNA and 1/10 volume of 3 M sodium acetate, pH 5.2. Pellets were washed with 1 ml 70% ethanol and dissolved in 30 μl TE. The encapsidated viral DNA was subjected to agarose gel electrophoresis, followed by denaturation and Southern blotting, and detected by hybridization with a ^32^P-labeled full-length WHV probe.

### Staining of cell surface markers and intracellular IFN-γ of mouse splenocytes and flow cytometric analysis

WHV-specific CD8^+^ and CD4^+^ T cells were detected by established methods described in previous publications [10, 20]. Splenocytes in 96-well U-bottom plates were re-stimulated with individual peptides at a concentration of 2 μg/ml in the presence of brefeldin A (eBioscience, San Diego, CA) at 4 μg/ml for 5 h. All antibodies were purchased from Biolegend (San Diego, CA). Splenocytes were stained with fluorescein isothiocyanate (FITC)-conjugated anti-CD8 and phycoerythrin (PE)-conjugated anti-CD4, while dead cells were excluded by 7-AAD staining (BD pharmingen, NJ). For intracellular IFN-γ staining, cells were permeabilized by Fixation & Permeabilization kit (eBioscience) and stained with allophycocyanin (APC)-conjugated anti-IFN-γ (Biolegend). Data were analyzed using FACS Calibur (BD Biosciences, San Jose, CA) and FlowJo software (Treestar, Ashland, OR).

### Statistical analysis

The statistical analysis was carried out using Statistical Package for Social Sciences (SPSS 11.0, Chicago, IL). Analysis of variance with independent samples t-test was used to determine significant differences in comparisons. P<0.05 was considered as statistically significant. Data are presented as means ± standard deviation.

## Results

### Persistence of WHV genome in mice after hydrodynamical injection (HI)

The prolonged HBV persistence after HI in mice depends on the specific backbone of the vector carrying HBV genome and the genetic background of the mouse strain. We asked whether WHV may maintain the gene expression and replication in the mouse model after HI. We have tested both C57BL/6 and BALB/c mice HI with pBS-WHV1.3 at the initial experiments. At day 10 after HI, we tested and compared the intrahepatic WHcAg expression and the serum WHV DNA. The percentages of WHcAg-expressing hepatocytes in BALB/c and C57BL/6 mice were comparable. However, the serum WHV DNA level was about 10^5^ copies/ml in BLAB/c mice, but lower to undetectable in C57BL/6 mice. Therefore, BALB/c mice were chosen for our present study. Ten μg of pBS-WHV1.3 containing a 1.3 fold overlength WHV genome were injected hydrodynamically in BALB/c mice. Mouse sera were taken at the indicated time points after HI and subjected to the detection of viral DNA by PCR. All mice were positive for serum WHV DNA at days 10 and 18 after HI. Surprisingly, serum WHV DNA remained detectable up to 40 weeks after HI in 6 of 9 mice, though the amount of PCR products decreased over time (Fig 1A). For the low viral load and the limited volume of available sera, the PCR detection of serum WHV DNA failed at some time points.

Consistent with the serum WHV DNA, the intrahepatic WHcAg and WHsAg were detected by ICS in all tested mice at day 10, 42, 98 after HI. At each time point, the liver samples of at least three mice were taken for ICS. About 4% of hepatocytes were positively stained for WHcAg at day 10 after HI. WHcAg-positive hepatocytes were randomly distributed through the liver lobules with a tendency for localization in the centrilobular area (Fig 1B). The number of WHcAg-positive hepatocytes and the intensity of ICS decreased with time. At days 42 and 98 after HI, about 2.9% and 1.2% of hepatocytes were WHcAg positive, respectively (Fig 1B). WHsAg expression showed the same pattern like WHcAg. At days 10, 42, and 98 after HI, the frequency of WHsAg-positive hepatocytes were 5%, 1%, and 1%, respectively (Fig 1B). The results indicated that after HI of pBS-WHV1.3, WHV genome may persist in mice and remain functional over months.

### HBsAg expression of the recombinant WHV-HBV genomes in transiently transfected hepatoma cells

**Fig 2:**
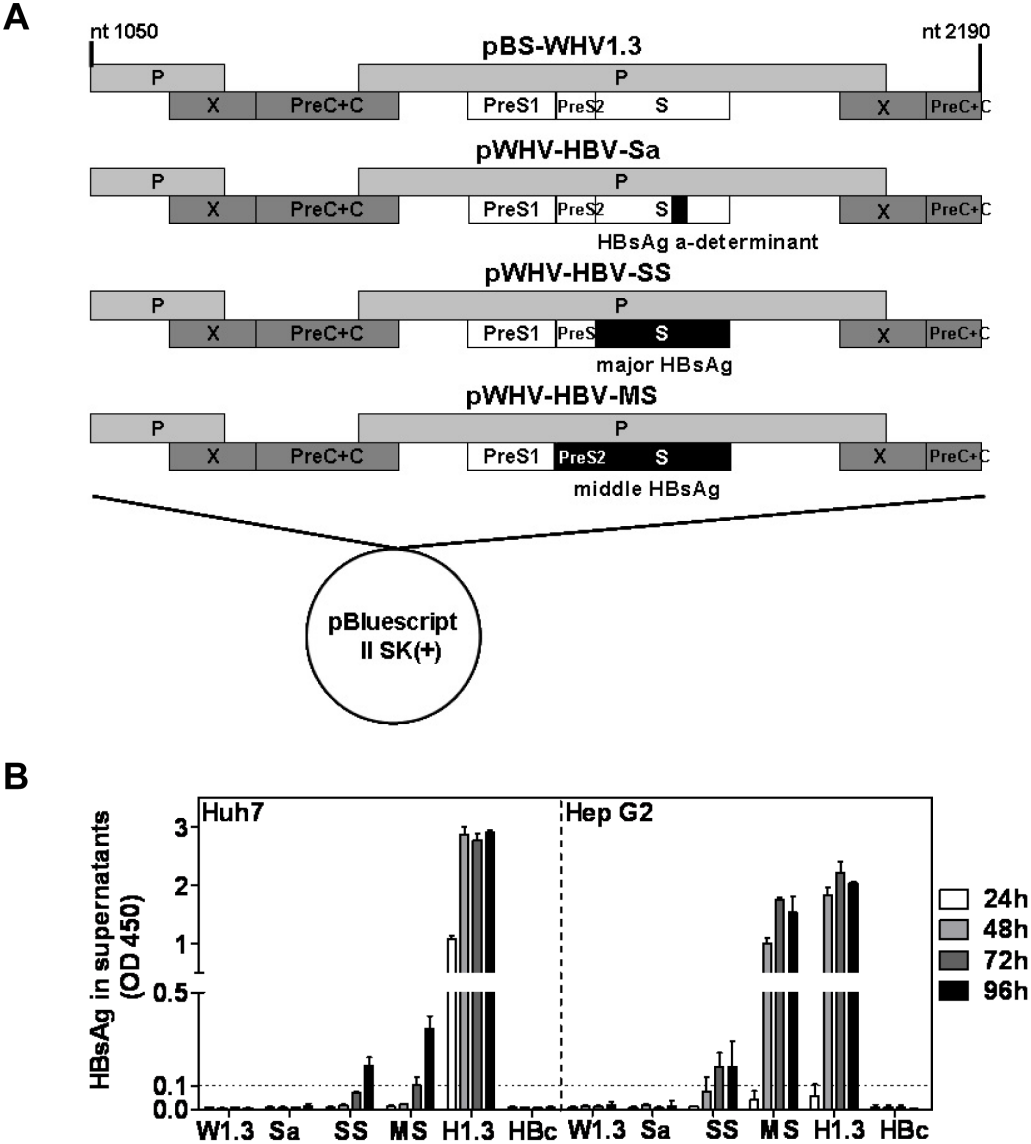
Constructions of recombinant WHV-HBV genomes and transient transfection of recombinant WHV-HBV genomes in hepatoma cells. (A) The schematic map of recombinant WHV-HBV genomes. pBS-WHV1.3 contained a 1.3 fold overlength WHV genome in pBluescript II SK(+) vector and was used as backbone. The respective WHV genome regions were replaced by the corresponding HBV sequences, shown as black bars. The plasmids pWHV-HBV-Sa, pWHV-HBV-SS, and pWHV-HBV-MS contained HBV sequences encoding HBsAg a-determinant only, major HBsAg and middle HBsAg, respectively. (B) HBsAg expression in the supernatant of the transfected hepatoma cells. Huh7 and HepG2 cells were transiently transfected with plasmids of pBS-WHV1.3 (W1.3), pWHV-HBV-Sa (Sa), pWHV-HBV-SS (SS), pWHV-HBV-MS (MS), pBS-HBV1.3 (H1.3), and pHBc (HBc). The culture supernatants were collected at 24, 48, 72, and 96 hours after transfection for the detection of HBsAg by ELISA. The results were read at OD 450 nm. The cut off value was set as 0.1 and indicated by the dotted line.

Further, we asked whether WHV genome carrying foreign DNA sequences could maintain the long term gene expression and persistence in mice after HI. For this purpose, the coding region of HBsAg was inserted into pBS-WHV1.3 to replace the corresponding WHV region. Based on pBS-WHV1.3, three plasmids pWHV-HBV-Sa, pWHV-HBV-SS, and pWHV-HBV-MS with HBV genome regions encoding HBsAg a-determinant only, major HBsAg, and middle HBsAg were constructed (Fig 2A). The respective regions of WHV genome in pBS-WHV1.3 were replaced by the corresponding HBV sequences. This procedure led to the substitutions in both amino acid sequences of WHV surface antigen and polymerase (S2 Fig). For pWHV-HBV-Sa, the coding region of WHsAg aa 117–143 was substituted by that of HBsAg a-determinant aa 121–147 [17]. Accordingly, pWHV-HBV-SS and pWHV-HBV-MS expressed major and middle HBsAg instead of WHsAg, respectively. To examine whether these plasmids were able to express chimeric WHsAg or complete HBsAg detectable by HBsAg immunoassay, Huh7 and HepG2 cells were transiently transfected with these plasmids and the control plasmids pBS-WHV1.3, pBS-HBV1.3, and pHBc (a plasmid expressing HBV core antigen) [4]. Culture supernatants of the transfected cells were collected for HBsAg analysis. The transfection with pWHV-HBV-SS, pWHV-HBV-MS, and pBS-HBV1.3 led to the production of detectable HBsAg in both hepatoma cell lines, though at very different levels (Fig 2B). Rather unexpectedly, the transfection with pWHV-HBV-Sa did not result in the production of chimeric surface antigen detectable in HBsAg immunoassay. Previously, the chimeric WHsAg with HBsAg a-determinant has been found to be reactive with antibodies to HBsAg (anti-HBs) [17] and induce anti-HBs in mice by vaccination [21]. pWHV-HBV-Sa may express the chimeric WHsAg at a very low level *in vitro* but at detectable levels *in vivo* (see below). To detect the replication competence of the chimeric genomes *in vitro*, we performed the Southern blot assay after the transfection in Huh7 cells. In general, the replication level of the chimeric genomes was low, so we needed a long exposure of blots to detect the replication intermediates (S3 Fig). The replication intermediates of woodchuck hepatitis virus do not form distinct bands like HBV, rather a smear with undefined sizes [22].

### Persistence of the recombinant genome WHV-HBV-Sa in mice after HI

**Fig 3:**
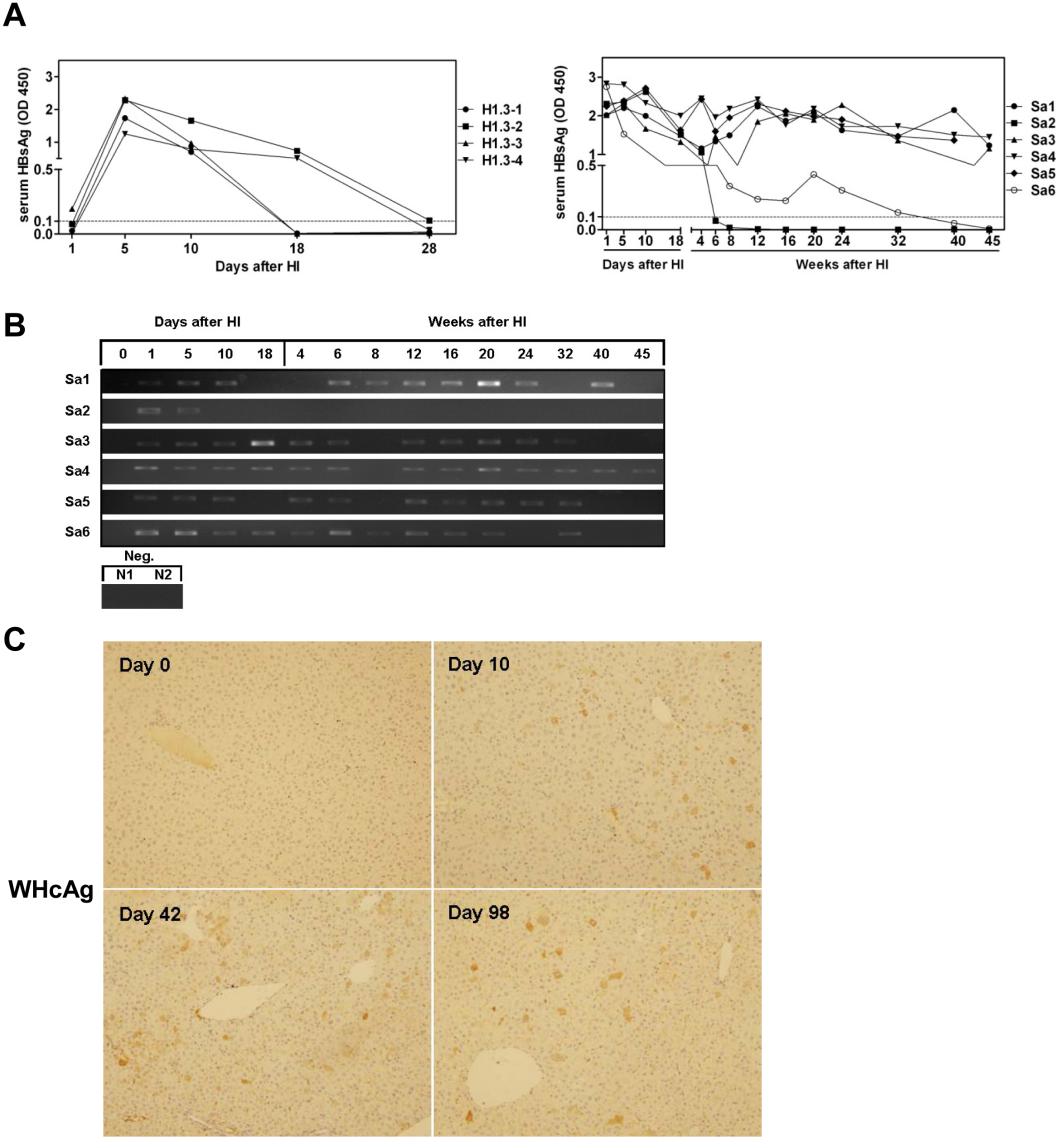
Serum viral DNA and the expression of viral proteins in mice after HI with pWHV-HBV-Sa. (A) Six mice (Sa1-Sa6) were hydrodynamically injected with pWHV-HBV-Sa, while four mice (H1.3–1 to H1.3–4) were hydrodynamically injected with pBS-HBV1.3. Mouse sera were collected at the indicated time points after HI and subjected to HBsAg ELISA. The results were read at OD 450 nm. The cut off value was set as 0.1 and indicated by the dotted line. (B) Detection of serum encapsidated viral DNA by PCR. PCR products were visualized by agarose gel electrophoresis. Neg.: Sera from two mice (N1, 2) challenged by saline. (C) Detection of hepatic WHcAg expression by ICS at days 0, 10, 42 and 98 after HI with pWHV-HBV-Sa. A set of representative samples was shown. Magnification: 200×.

Six BALB/c mice Sa1-Sa6 were challenged by HI with pWHV-HBV-Sa, while four mice (H1.3–1 to H1.3–4) were hydrodynamically injected with pBS-HBV1.3. Mouse sera were taken at the indicated time points after HI and subjected to serological assays for HBsAg and anti-HBs, and PCR detection of encapsidated viral DNA. All 4 mice after HI with pBS-HBV1.3 were HBsAg positive at day 5 and turned to HBsAg negative at day 28 after HI, consistent with the previous studies (Fig 3A). The serum samples from 4 mice (Sa1, 3, 4, 5) were highly positive in HBsAg ELISA from day 1 on and up to week 45 after HI. The serum samples from mouse Sa2 became negative in HBsAg ELISA from week 6 on. Another mouse Sa6 was positive in HBsAg ELISA until week 32 (Fig 3A). None of the six mice was positive for anti-HBs. The PCR results confirmed the persistence of viral DNA in mouse sera at low levels up to week 45, and were well correlated with HBsAg ELISA results (Fig 3B). ICS showed that WHcAg was expressed in 3.2%, 2.3%, and 1.4% of hepatocytes at days 10, 42, and 98 after HI, respectively (Fig 3C). In addition, WHcAg expression was detectable in the liver of some mice even at week 45, which was well consistent with the presence of chimeric surface antigen produced in serum (S4 Fig, S3 Table). These results indicated the long term persistence of the recombinant genome of WHV-HBV-Sa and gene expression after HI in the mouse model.

**Fig 4:**
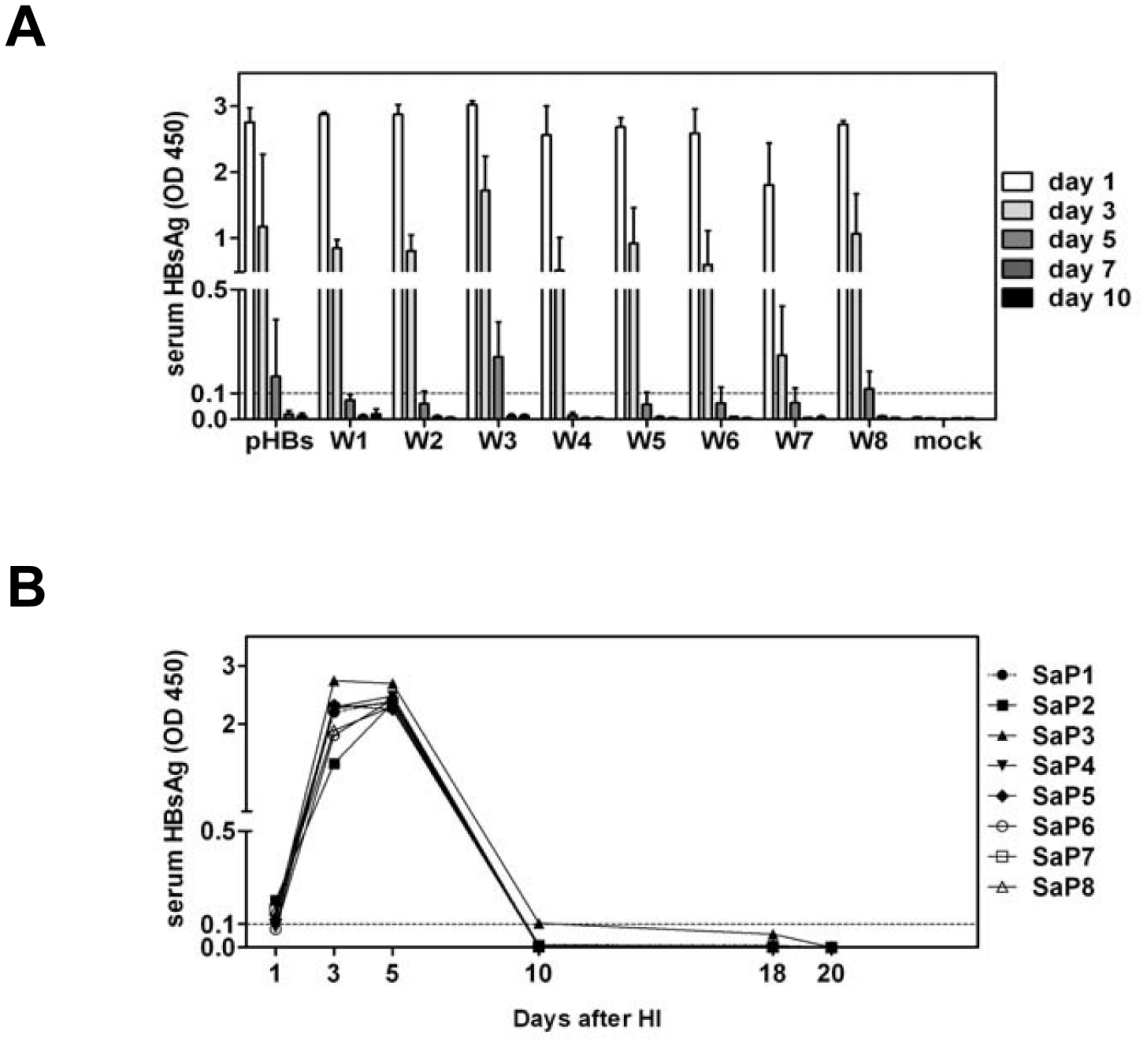
Detection of the surface antigen expression in mouse sera by HBsAg ELISA after HI with pHBsW1-8 and pSaΔP. Mouse sera were collected at the indicated time points after HI and subjected to HBsAg ELISA. (A) Each group with three mice was hydrodynamically injected with pHBsW1-8 (W1-W8). Mice injected with pHBsBK (pHBs) and saline (mock) were used as positive and negative control, respectively. (B) Eight mice (SaP1-8) were hydrodynamically injected with the mutated pWHV-HBV-Sa, pSaΔP. The results were read at OD 450 nm. The cut off value was set as 0.1 and indicated by the dotted lines.

The results above raised the questions why pBS-WHV1.3 and pWHV-HBV-Sa could persist in mice after HI. We asked whether partial WHV genome fragments may support the prolonged HBsAg expression in mouse after HI. To answer the question, WHV genome was dissected in 8 overlapping fragments by PCR and cloned into pHBsBK, a HBsAg expression plasmid based on pcDNA3, resulting in a set of plasmids of pHBsW1-W8 (S5 Fig). HI with pHBsW1-W8 led to the transient expression of HBsAg in mouse sera. However, serum HBsAg disappeared quickly at days 3 to 5 after HI (Fig 4A). Therefore, the prolonged gene expression *in vivo* was not determined by a partial fragment of WHV genome tested so far.

It is also possible that the prolonged viral gene expression may be due to the persistence of the residual plasmid DNA after HI. To test this possibility, pSaΔP, harboring a mutated start code of WHV polymerase in pWHV-HBV-Sa, was constructed and hydrodynamically injected in eight BALB/c mice (SaP1-8). The chimeric WHsAg with HBV a-determinant in mouse sera detected by HBsAg ELISA peaked at day 5 and disappeared at day 10 after HI (Fig 4B). The encapsidated viral DNA in serum was not detectable. This result demonstrated that the prolonged viral gene expression was not produced by the residual plasmid DNA, but required the replication of WHV and the recombinant WHV-HBV-Sa genome *in vivo*.

### Persistence of the recombinant genomes of WHV-HBV-SS and WHV-HBV-MS in mice after HI

**Fig 5:**
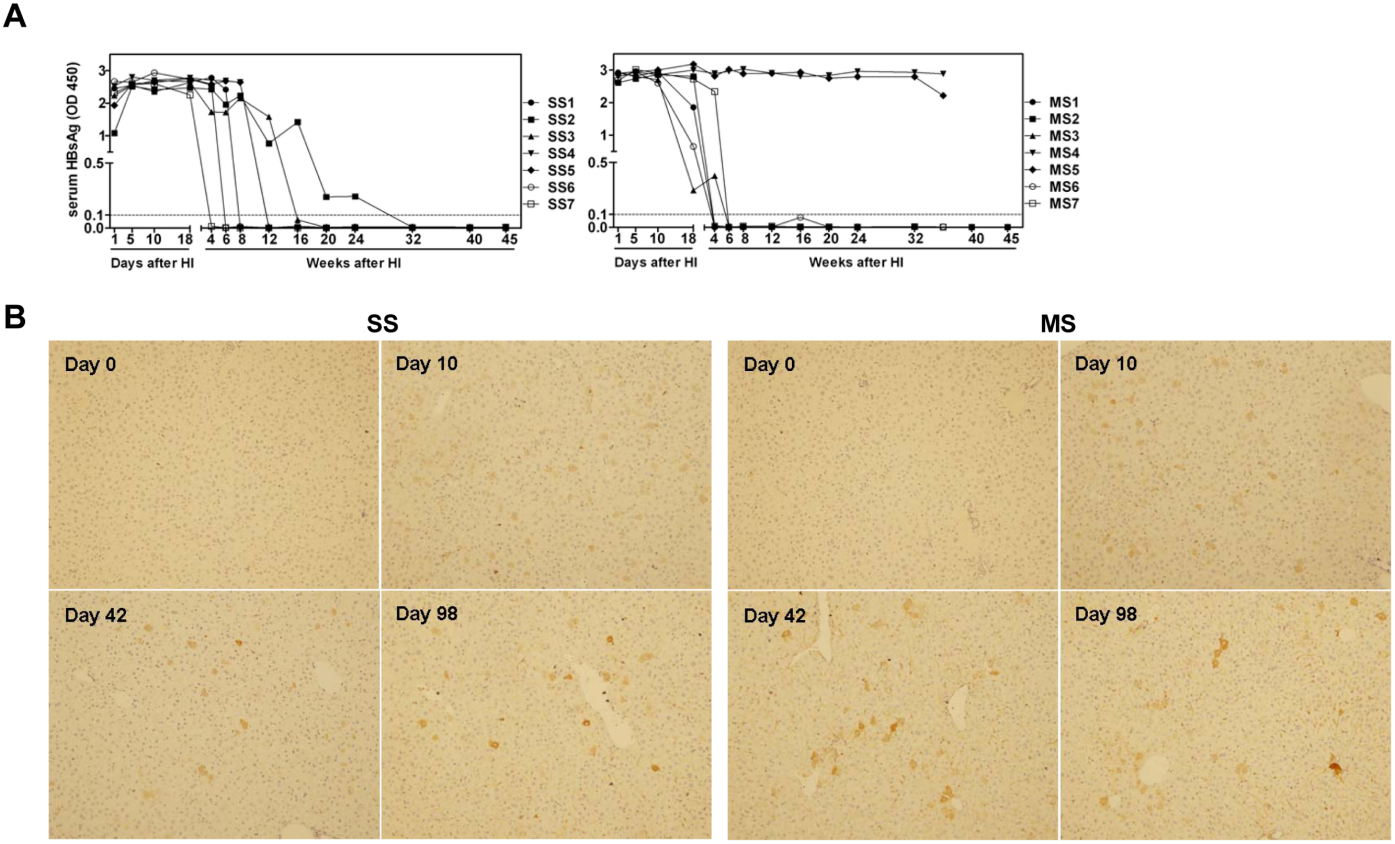
Serum HBsAg and intrahepatic WHcAg expression in mice after HI with pWHV-HBV-SS and pWHV-HBV-MS. (A) Mouse sera were collected after HI with pWHV-HBV-SS (SS) and pWHV-HBV-MS (MS) at the indicated time points and subjected to HBsAg ELISA. SS1-7 and MS1-7 indicated seven individual mice each group. In MS group, two mice of MS4 and MS5 were sacrificed at week 36 after HI, and the mouse splenocytes were taken for ELISpot assay and flow cytometry. The ELISA results were read at OD 450 nm. The cut off value was set as 0.1 and indicated by the dotted lines. (B) Mouse liver samples taken at the indicated time points after HI were detected for WHcAg expression by ICS. A set of representative samples was shown. Magnification: 200×.

Besides pWHV-HBV-Sa, the recombinant constructs of pWHV-HBV-SS and pWHV-HBV-MS were injected hydrodynamically into BALB/c mice. The HBsAg levels and the viral DNA in mouse sera were examined at the indicated time points after HI by HBsAg ELISA and PCR. The mice received pWHV-HBV-SS and pWHV-HBV-MS showed significantly prolonged HBsAg antigenemia than that with pBS-HBV1.3 and lost HBsAg positivity gradually (Fig 5A). Notably, two mice with pWHV-HBV-MS (MS4 and MS5) were continuously HBsAg positive at high levels until week 36 after HI. So far tested, no anti-HBs was detectable in mice receiving chimeric WHV-HBV genomes. Meanwhile, the mouse liver samples were subjected to ICS. The frequencies of WHcAg positive hepatocytes at days 10, 42, and 98 after HI with pWHV-HBV-SS were 4.3%, 1.8%, and 1.3%, respectively (Fig 5B). In pWHV-HBV-MS injected mice, the frequencies of WHcAg expressing hepatocytes were 4.1%, 1.5%, and 1% at days 10, 42, and 98 after HI, respectively (Fig 5B). Hepatic WHcAg expression at week 45 after HI was also examined by ICS. The results were consistent with the detection of serum HBsAg (S4 Fig and S3 Table). In addition to the hepatic WHcAg expression, HBsAg expressions in mouse livers at day 10 and 42 after HI were also detectable by ICS (S6 Fig). The persistent expression of hepatic WHcAg and HBsAg confirmed that chimeric viral genomes remain functional for a prolonged period in mice after HI. Similar to the previous experiments, the encapsidated vial DNA in sera was detected at low levels by PCR (S6 Fig). However, the presence of HBsAg and viral DNA in sera was not well correlated.

### Replication of WHV and the recombinant WHV-HBV genomes in mouse after HI

**Fig 6:**
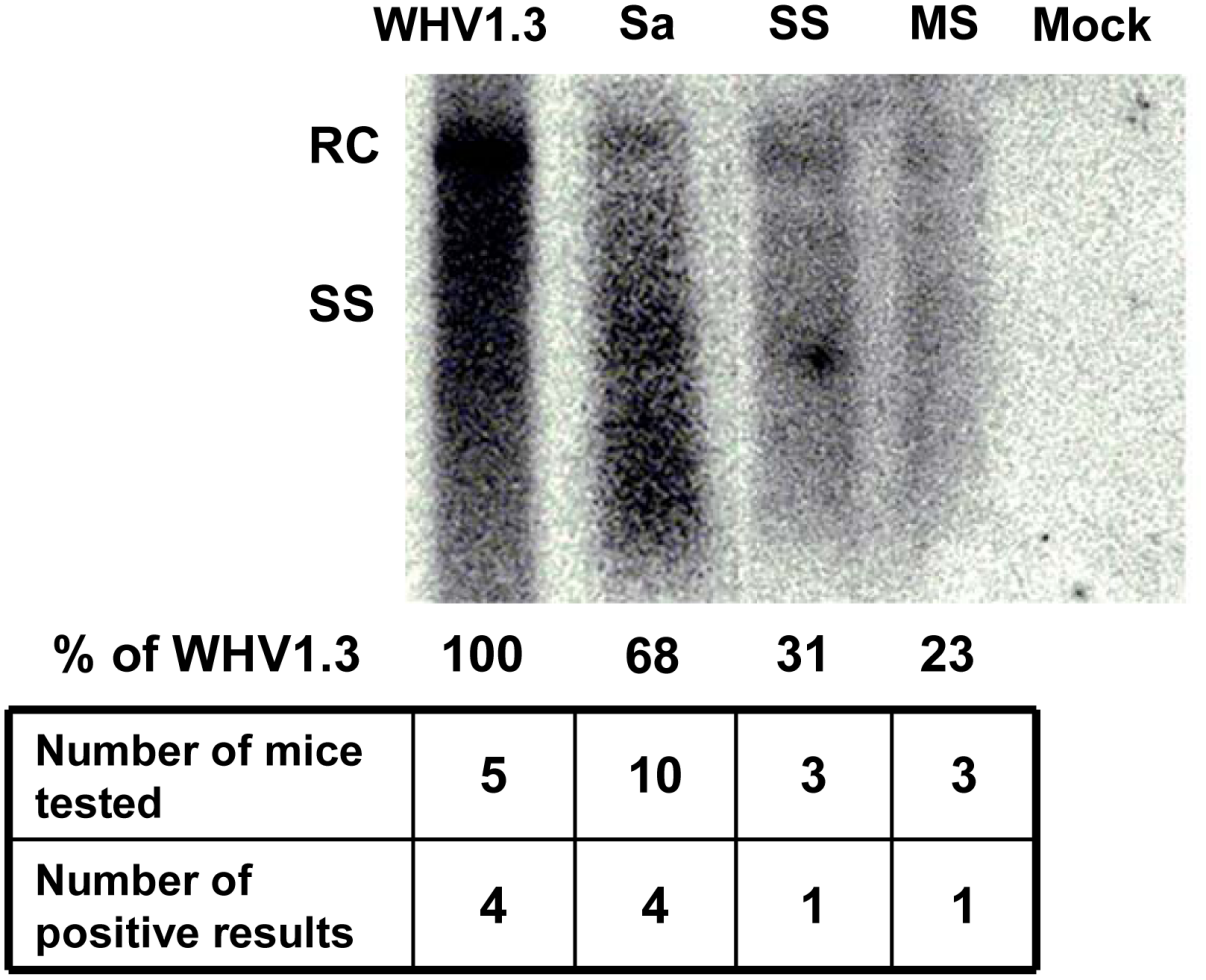
Detection of the replication intermediates of WHV and the recombinant WHV-HBV genomes in mouse liver. Mouse liver samples were collected at day 10 after HI with pBS-WHV1.3 (WHV1.3), pWHV-HBV-Sa (Sa), pWHV-HBV-SS (SS), pWHV-HBV-MS (MS) and saline (Mock). The replication intermediates were detected by Southern blot using a full length WHV genome as probe. The signal intensity was quantified by measuring the grey scale using WHV1.3 as the reference. RC DNA: relaxed circular DNA; SS DNA: single stranded DNA.

We detected the replication intermediates in mouse liver after HI with pBS-WHV1.3 and recombinant constructs. Mouse liver tissue was collected at day 10 after HI and the encapsidated viral DNA was analyzed by Southern blot to detect the replication intermediates. The replication intermediates including relaxed circular DNA (RC) and single stranded DNA (SS) were detectable in mouse liver (Fig 6). However, it indicated the low levels of viral replication *in vivo*, and not all the samples were positive. We found that the recombinant genomes of WHV-HBV-SS and WHV-HBV-MS were of much weaker replication competence after HI in mice, compared with the genome of WHV and WHV-HBV-Sa. The replacement of long fragment in the recombinant genomes may influence the function of polymerase and reduced the replication competence.

### Specific T cell responses to WHV and HBV antigens in mice after HI

**Fig 7:**
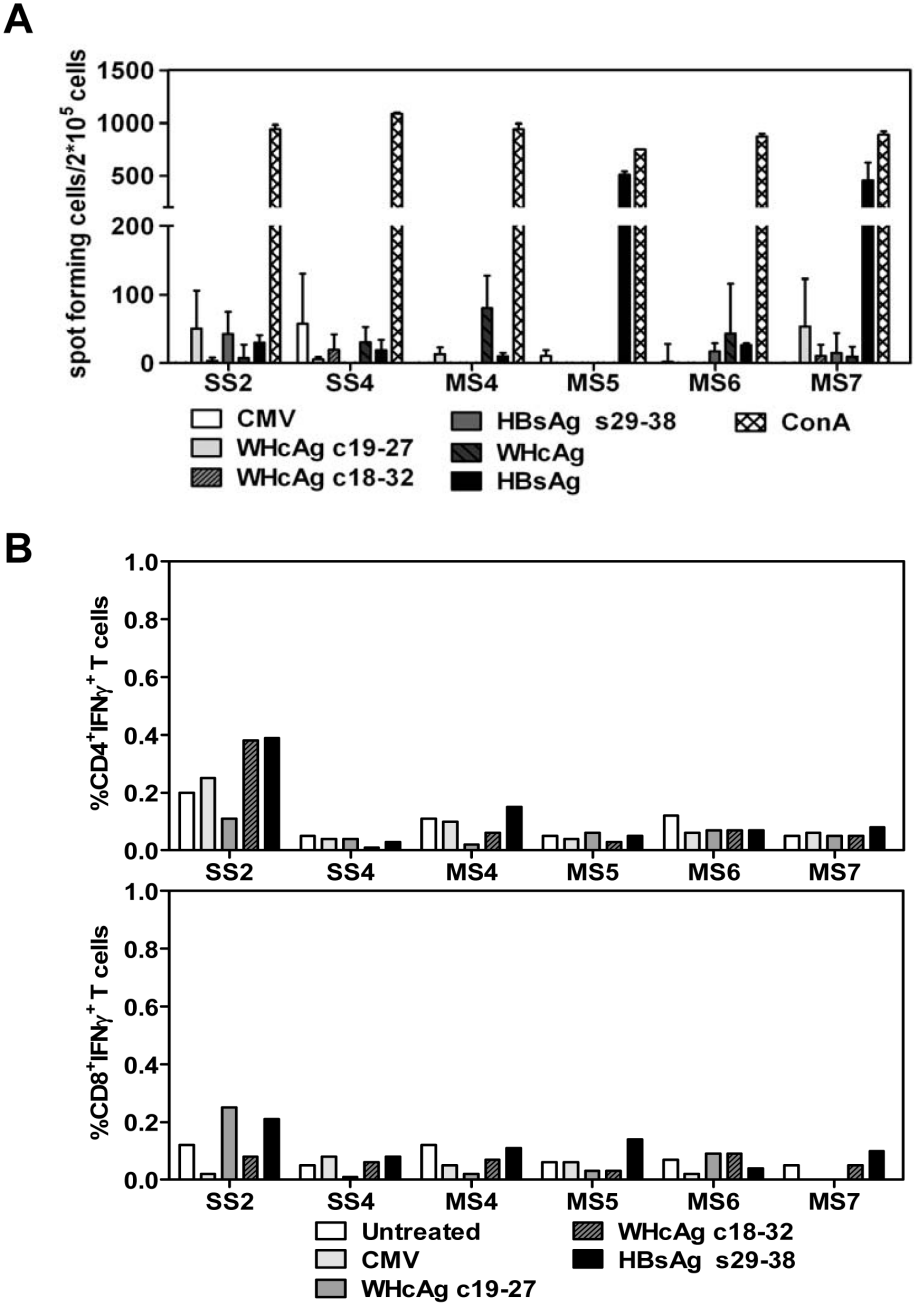
The specific T cell responses to WHcAg and HBsAg in BALB/c mice received HI with the recombinant WHV-HBV genomes. Mouse splenocytes were taken at week 36 after HI with pWHV-HBV-SS (SS) and pWHV-HBV-MS (MS). The splenocytes were cultured in presence of HBsAg-derived peptide s29-38, WHcAg-derived peptides c19-27 and c18-32, WHcAg, and HBsAg. (A) IFN-γ producing cells were detected by ELISpot assay. The results were shown as spot-forming cells per 2×10^5^ cells. ConA and an unrelated CMV peptide were used as positive and negative control, respectively. (B) The frequencies (%) of IFN-γ producing CD4^+^ and CD8^+^ T cells in mouse splenocytes were analyzed by staining of cell surface markers and intracellular IFN-γ and flow cytometry. Untreated: splenocytes cultured without specific peptides.

The specific T cell responses determined the outcome of HBV genome after HI in mouse model [1]. Both WHcAg and HBsAg have been suggested to be the major targets of host immune responses and play a critical role in viral clearance [23–25]. Therefore, specific T cell responses to WHcAg and HBsAg were measured in some BALB/c mice hydrodynamically injected with pWHV-HBV-SS and pWHV-HBV-MS. Mouse splenocytes were prepared at week 36 after HI and subjected to ELISpot assay for the detection of WHcAg- and HBsAg-specific IFN-γ-producing cells. Further, mouse splenocytes were cultured in the presence of selected WHcAg- and HBsAg-derived peptides to expand specific CTLs *in vitro*. The frequencies of CD4^+^ IFN-γ^+^ T cells and CD8^+^ IFN-γ^+^ T cells in splenocytes were analyzed by staining of cell surface markers and intracellular IFN-γ and flow cytometry. However, the positive response in ELISpot assay to HBsAg was only seen in mice MS5 and MS7 (Fig 7). Otherwise, both methods failed to demonstrate the presence of specific T cells to WHcAg and HBsAg in splenocytes from other mice (Fig 7). These results suggested that HI with recombinant WHV-HBV genomes primed only limited specific T cell responses against WHV and HBV proteins in BALB/c mouse.

## Discussion

In the present study, we found that WHV and the recombinant WHV-HBV genomes were able to persist and express WHV and HBV proteins in mice after HI for a prolonged period of time. The recombinant genome of WHV-HBV-Sa with HBsAg a-determinant in WHsAg backbone expressed the chimeric surface antigen at relative high levels for over 40 weeks. Both viral DNA and antigens in mouse sera and liver samples were consistently detectable. The recombinant genomes of WHV-HBV-SS and WHV-HBV-MS with the substitution of the complete coding regions of small and middle HBsAg showed significantly prolonged HBsAg antigenemia. Serum HBsAg and hepatic WHcAg expression were well correlated.

Previously, it was reported that HI of an overlength HBV genome in an AAV vector could lead to persistent HBV replication and gene expression in C57BL/6 mice [1]. Both the vector backbone and the host genetic background were found to be important for HBV persistence [1]. In our study, the 1.3 fold overlength WHV genome and the recombinant WHV-HBV genomes cloned in pBluescript II SK(+) vector could persist in BALB/c mice after HI. However, the pBluescript II SK(+) vector does not support the long term persistence of 1.3 fold overlength HBV genome in BALB/c mice. Thus, we attempted to determine which part of WHV genome might cause the persistent gene expression in mice. However, the partial WHV genome fragments were not able to maintain HBsAg expression in mice, as shown in Fig 4A. In addition, the mutated recombinant WHV-HBV genome of pSaΔP without replication competence could not support the long-term antigen expression *in vivo* either (Fig 4B). Thus, we concluded that a replication competent WHV genome is required to maintain the long-term gene expression and the persistence of viral DNA in mice after HI. In this case, the formation of functional WHV cccDNA in the mouse liver was presumed. This hypothesis has been discussed since the establishment of the hydrodynamic injection mouse model. It has been established that cccDNA is present at exceedingly low or undetectable levels in hydrodynamically transfected mice [3], and even in HBV transgenic mice with high replication levels [26]. This is due to the existence of a species restriction on the production of cccDNA [27]. In our study, only very few hepatocytes (<10%) in the mice after HI with WHV and the recombinant genomes were positive for WHcAg expression and WHV replication. Therefore, no direct evidence for the WHV cccDNA in the mouse liver could be obtained currently by available methods.

Meanwhile, we found that HI of WHV and the recombinant WHV-HBV genomes did not trigger effective antibody and T-cell responses to viral proteins in BALB/c mouse. This is similar to the situation if pAAV/HBV1.2 has been used for HI in BL6/C57 mice [1]. The reasons of the weak immune responses to HI with WHV and WHV-HBV genomes in mice might be the low replication activity and the relative low expression levels of viral proteins. As these proteins are expressed in the liver, an immune-privileged organ, there is no effective immune activation after HI. WHV-specific immune responses, if pre-primed by vaccination, could clear WHV from mice after HI [20].

The primary goal of this study is to investigate whether WHV genome could replicate or maintain gene expressions in mice after hydrodynamic injection. Besides that, it was also considered to generate the replication competent WHV genome carrying the relevant HBV genes, which might be used for infection in woodchucks. The woodchuck model is an informative model for studies on HBV infection, including host immune responses and immunotherapies of chronic HBV infection. Unfortunately, the specific reagents like vaccines for HBV research could not be directly used in woodchucks. In an early study, it was found that the co-expression of HBV core antigen and WHcAg led to the production of capsid containing both components [28]. Defective HBV polymerase due to a insertion mutation could be complemented by WHV polymerase, allowing HBV replication, and vice versa [28]. HBV PreS region could be replaced by the corresponding region from WHV genome and did not affect the expression of HBV surface antigen (HBsAg) and assembly of virus particles [29]. In our previous experiments, a number of chimeric plasmids containing the complementary parts of HBsAg and WHsAg were constructed. Such recombinant plasmids are able to produce surface antigens and retained the correct antigenicity [17, 21]. In the present study, we have constructed and tested the various WHV-HBsAg recombinant genomes based on the replicable overlength WHV genome. We found that WHV genome could tolerate the replacement of the surface region by the corresponding sequences from the HBV genome. It will be interesting to investigate the replication of the recombinant genomes with complementary HBV and WHV sequences *in vivo*. In addition, our findings indicated the possibility to explore the overlength WHV genome for the liver-specific, long-term gene expression *in vivo*, if the desired sequence could be integrated into the WHV genome properly [30,31].

Taken together, the recombinant WHV and WHV-HBV genomes could persist and maintain the long term protein expression in mice. The ability of the recombinant WHV constructs to persist in mice is an interesting aspect for the future investigation and may be explored for *in vivo* gene transfer. In addition, our data suggests that the viral factor is an independent determinant for viral persistence in the mouse model, besides the vector backbone and host genetic background.

## Supporting Information

S1 Fig The schematic procedure of the construction of pWHV-HBV-SS and pWHV-HBV-MS. (TIF)

S2 Fig Alignment of amino acid sequences of the middle surface antigen (A) and polymerase (B) encoded by the wild type WHV and the recombinant genomes of WHV-HBV-Sa (Sa), WHV-HBV-SS (SS), WHV-HBV-MS (MS). (TIF)

S3 Fig Detection of the replication intermediates of WHV and chimeric genomes in the transfected Huh7 cells by Southern blot assay. (TIF)

S4 Fig WHcAg expression in mouse liver at week 45 after HI. (TIF)

S5 Fig Construction of pHBsW1-W8. (TIF)

S6 Fig Detection of viral DNA in mouse sera and hepatic HBsAg expression by ICS after HI with pWHV-HBV-SS (SS) and pWHV-HBV-MS (MS). (TIF)

S7 Fig The serum viral DNA levels were determined by real time PCR. (TIF)

S1 Table Primers used for PCR detection of chimeric viral DNA in serum. (DOC)

S2 Table Primers used for the construction of pWHV-HBV-SS, pWHV-HBV-MS and the mutated pWHV-HBV-Sa (pSaΔP). (DOC)

S3 Table Detection of viral DNA in sera, serum HBsAg, and hepatic WHcAg expression in mice received HI with pWHV-HBV-Sa, pWHV-HBV-SS, and pWHV-HBV-MS at week 45. (DOC)
