# Supplementary figures and images for: Persistence of the Recombinant Genomes of Woodchuck Hepatitis Virus in the Mouse Model
Source: PLoS One. 2015 May 5;10(5):e0125658. doi: 10.1371/journal.pone.0125658 (PMC4420481; doi:10.1371/journal.pone.0125658)

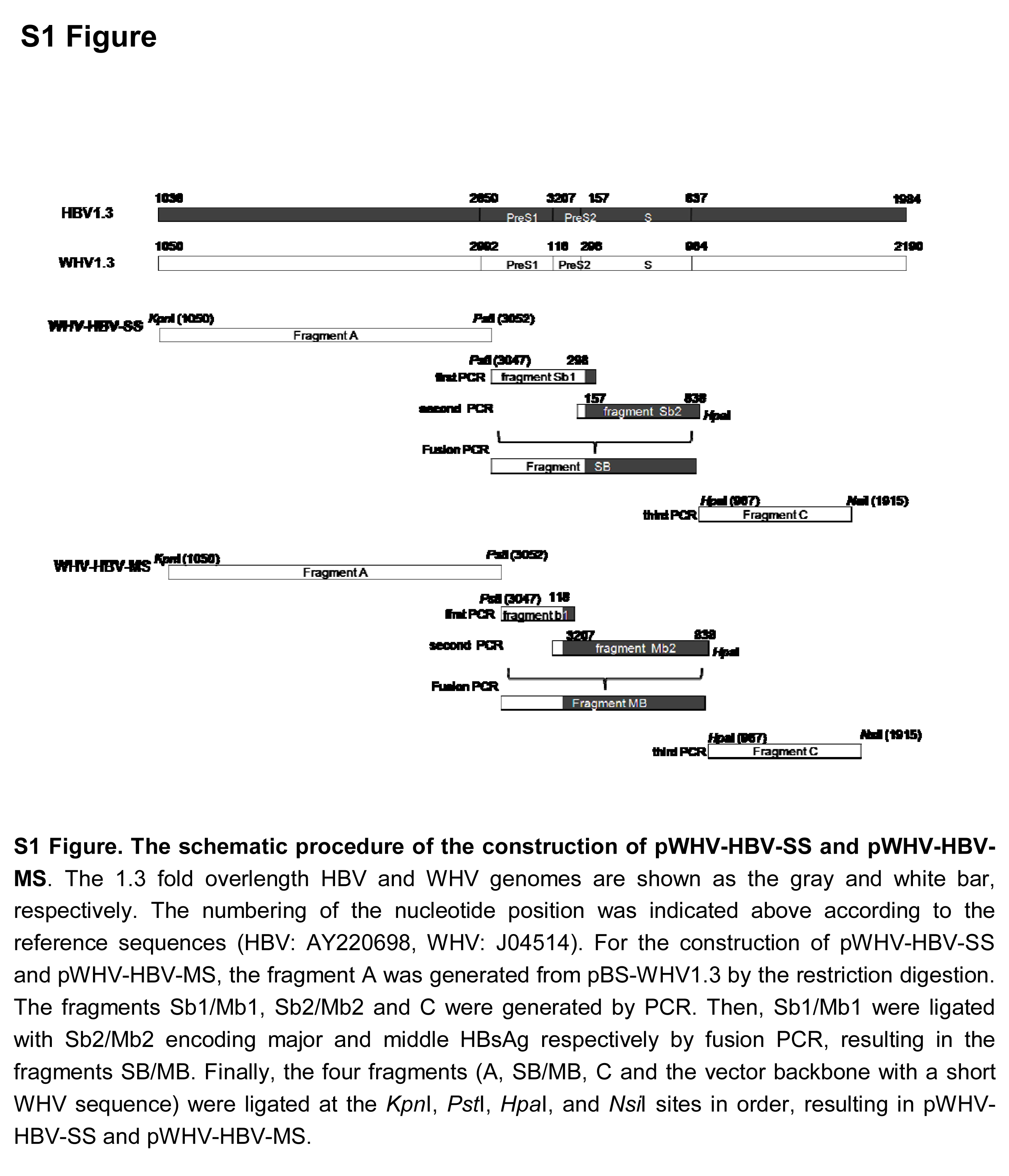

Supplement: S1 Fig — (TIF) [file pone.0125658.s001.tif]

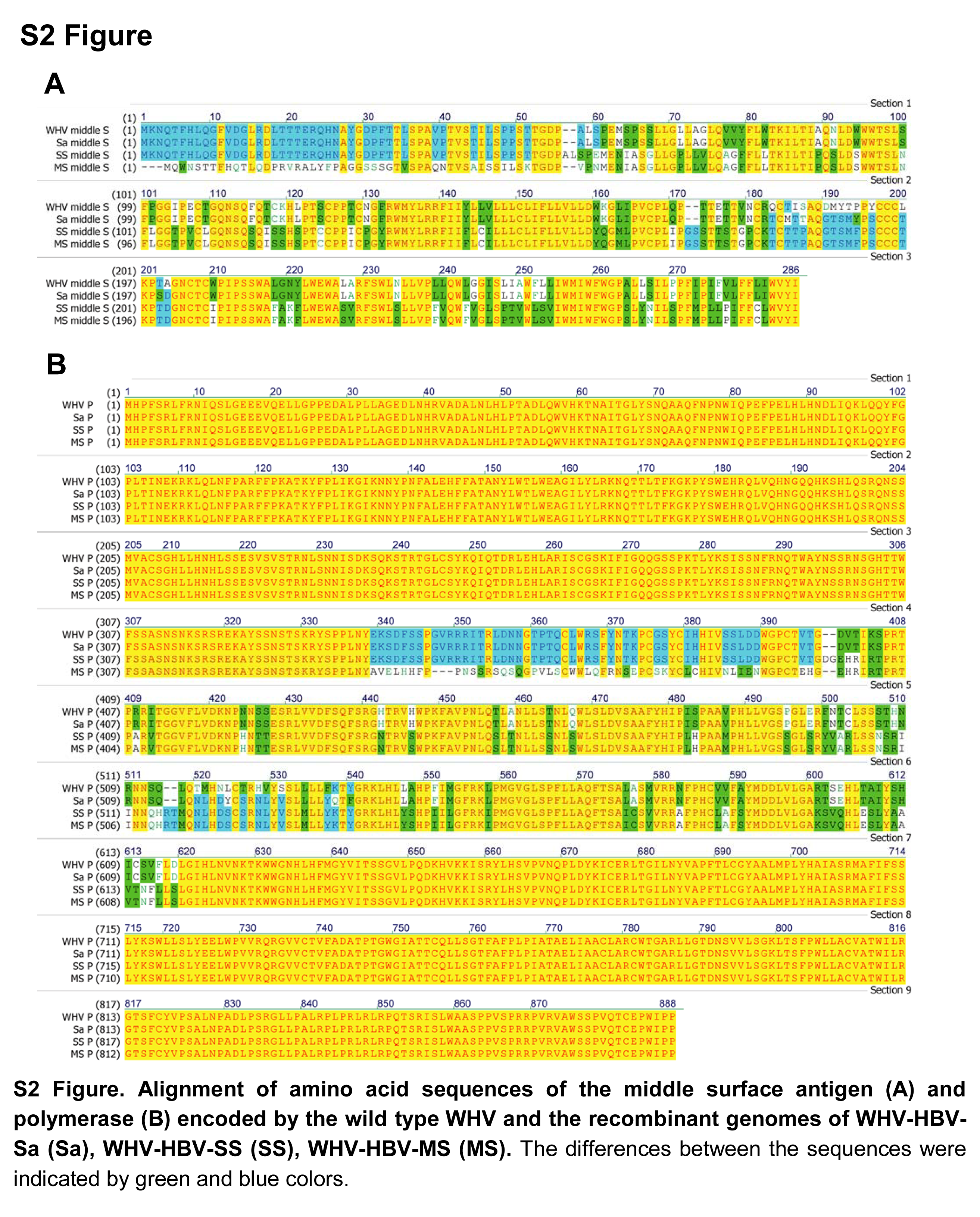

Supplement: S2 Fig — (TIF) [file pone.0125658.s002.tif]

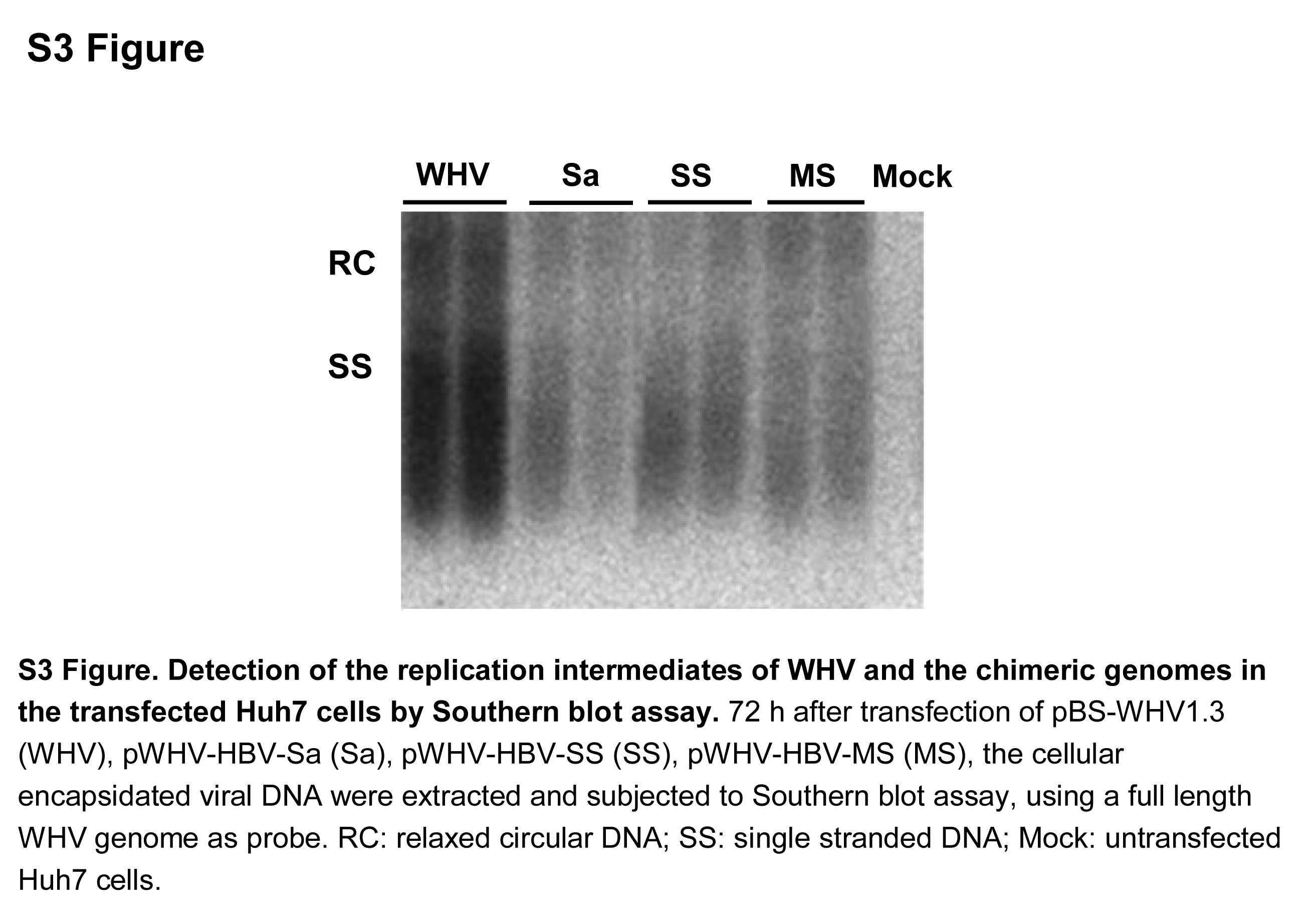

Supplement: S3 Fig — (TIF) [file pone.0125658.s003.tif]

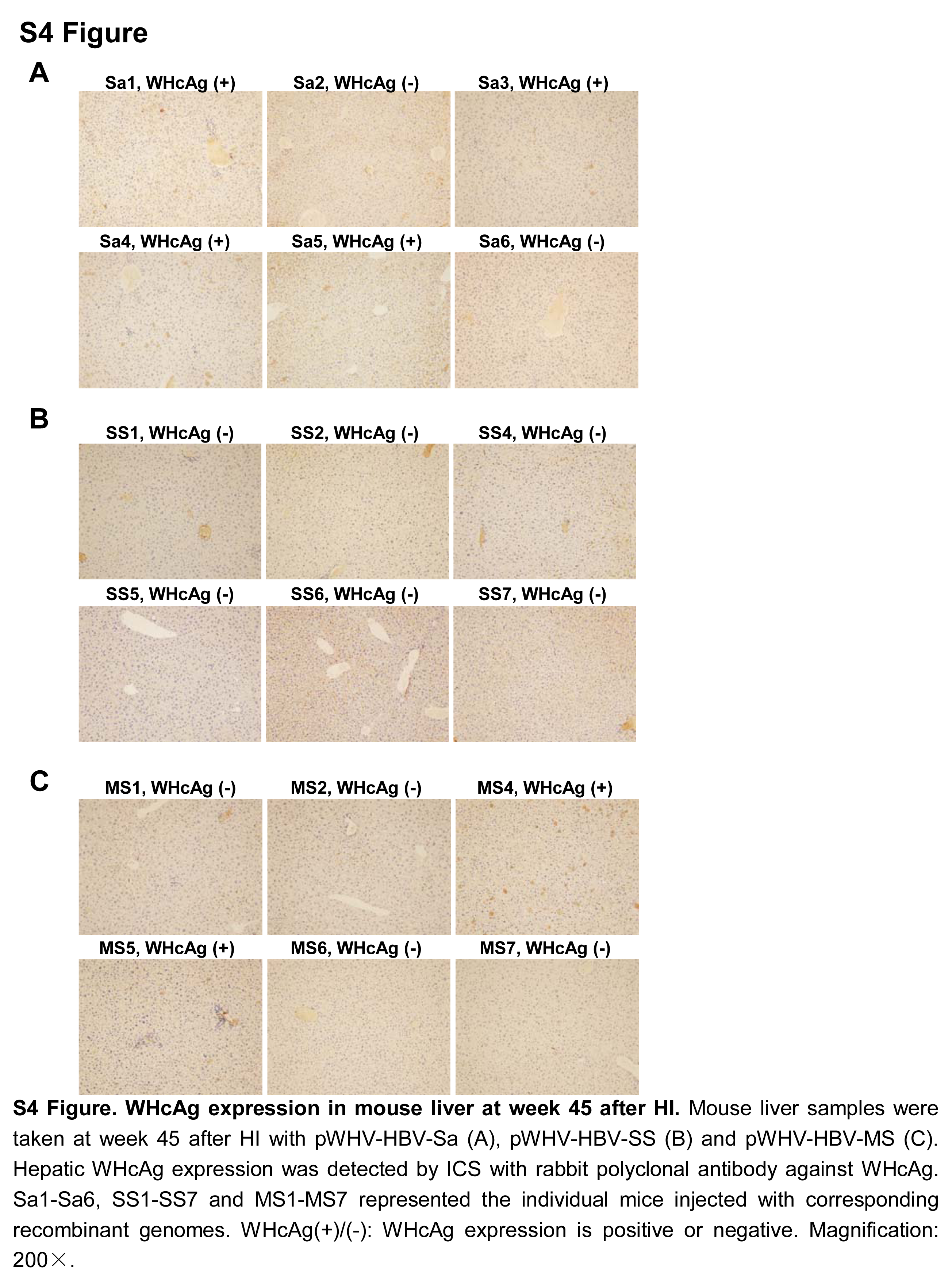

Supplement: S4 Fig — (TIF) [file pone.0125658.s004.tif]

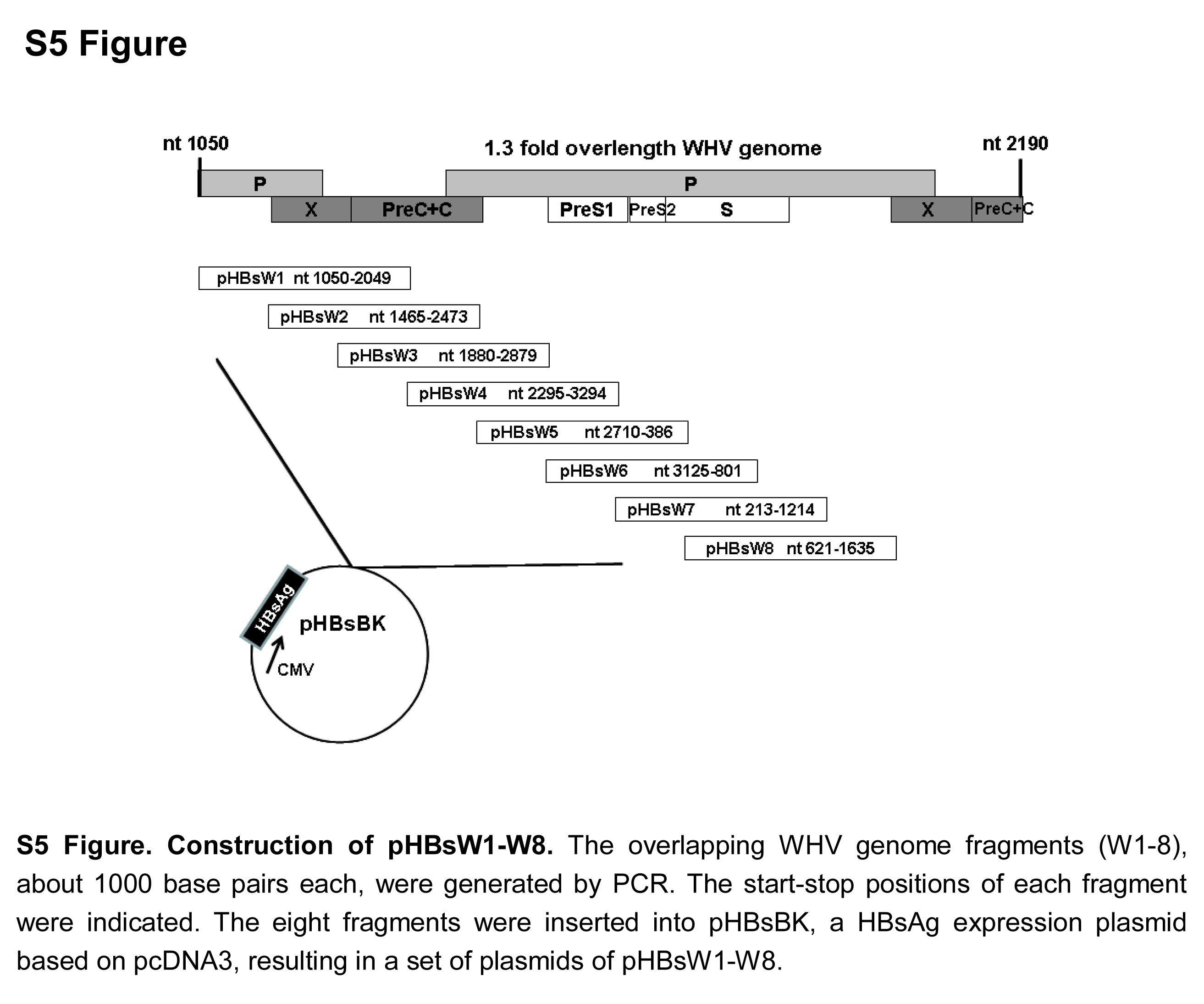

Supplement: S5 Fig — (TIF) [file pone.0125658.s005.tif]

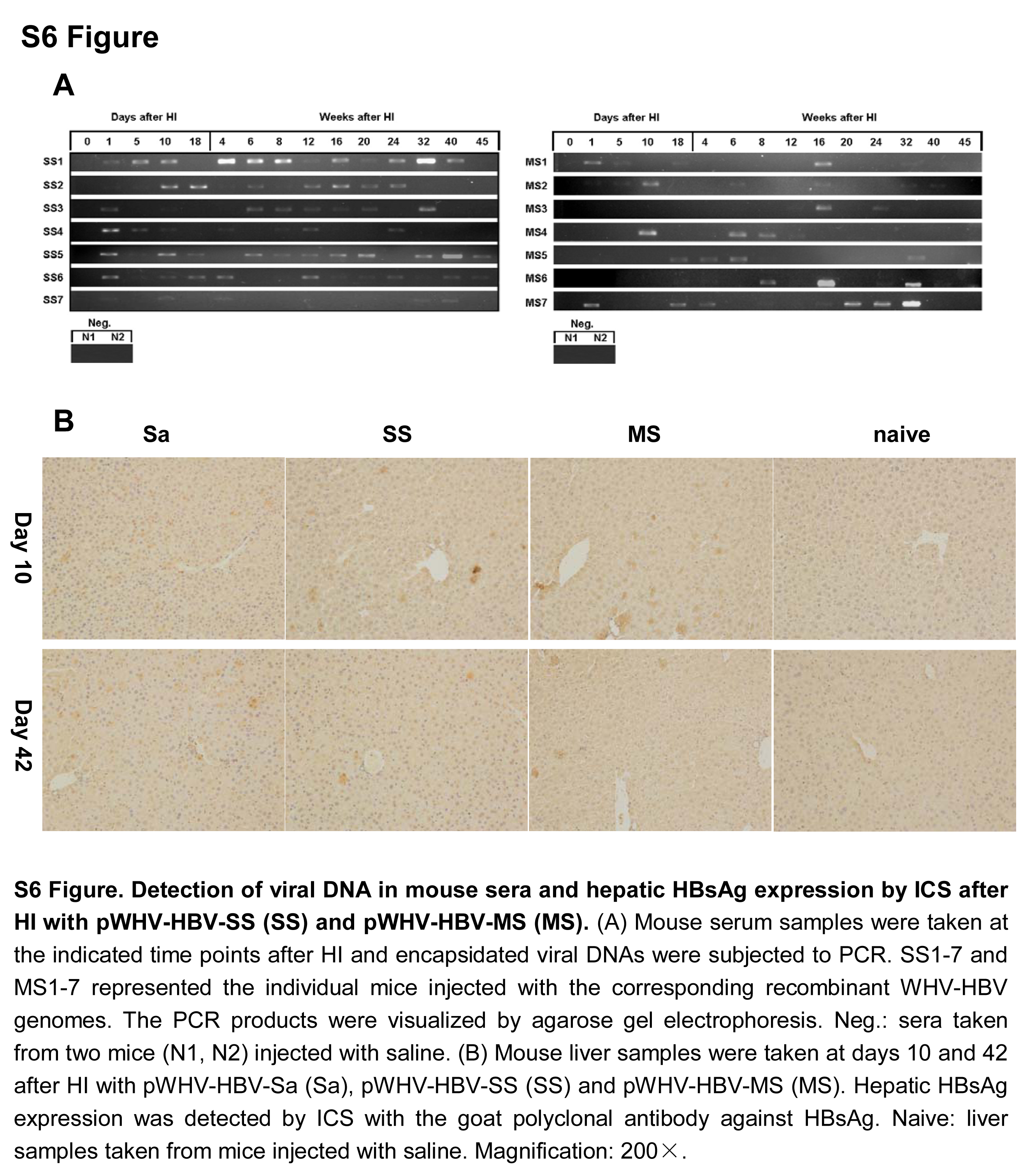

Supplement: S6 Fig — (TIF) [file pone.0125658.s006.tif]

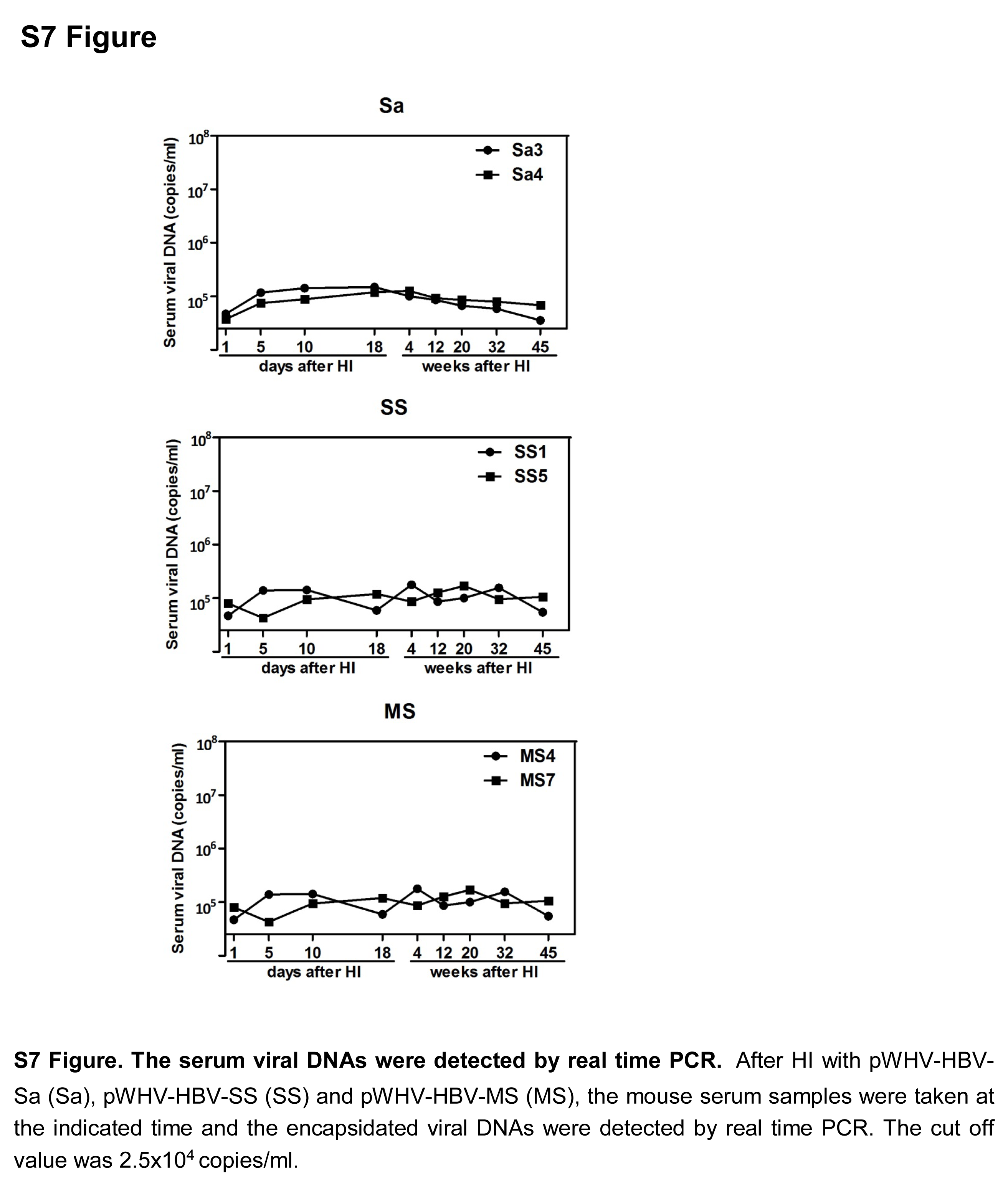

Supplement: S7 Fig — (TIF) [file pone.0125658.s007.tif]
