# Supplementary material for: Persistence of the Recombinant Genomes of Woodchuck Hepatitis Virus in the Mouse Model
Source: PLoS One. 2015 May 5;10(5):e0125658. doi: 10.1371/journal.pone.0125658 (PMC4420481; doi:10.1371/journal.pone.0125658)
Supplement: S1 Table — (DOC) [file pone.0125658.s008.doc]

**S1 Table.** Primers used for PCR detection of viral DNA in serum.

| **Designation** | **Polarity** | **Sequence** | **Position of 5’base** |
| --- | --- | --- | --- |
| WQp1 | Sense | gctttcgttggatgta | 501^a^ |
| WQp2 | Antisense | gaataaatggcggtaa | 908^a^ |
| HQp1 | Sense | tcttcatcctgctgctatgc | 407^b^ |
| HQp2 | Antisense | aaccactgaacaaatggcac | 704^b^ |

^a^ The numbering of the WHV genome is according to the Genbank accession no. J04514.

^b^ The numbering of the HBV genome is according to the Genbank accession no. AY220698.
