# Supplementary material for: Persistence of the Recombinant Genomes of Woodchuck Hepatitis Virus in the Mouse Model
Source: PLoS One. 2015 May 5;10(5):e0125658. doi: 10.1371/journal.pone.0125658 (PMC4420481; doi:10.1371/journal.pone.0125658)
Supplement: S2 Table — (DOC) [file pone.0125658.s009.doc]

**S2 Table**. **Primers used for the construction of pWHV-HBV-SS, pWHV-HBV-MS and the mutated pWHV-HBV-Sa (pSaΔP).**

| **Designation** | **Polarity** | **Sequence** | **Position**  **of 5’ base** |
| --- | --- | --- | --- |
| Sb1F | Sense | 5’- aaaactgcagtgggcacttatt-3’ | 3047^a^ |
| Sb1R | Antisense | 5’- tgatgcgatgttctccatctccggtgacagtgca -3’ | 298^a^ |
| Sb2F | Sense | 5’- tgcactgtcaccggagatggagaacatcgcatca -3’ | 150^b^ |
| Sb2R | Antisense | 5’- tc**gttaac**atttaaatgtatacccaaa -3’ | 838^b^ |
| C-F* | Sense | 5’- tc**gttaac**aaaacaaaatggtggggc -3’ | 967^a^ |
| C-R | Antisense | 5’- tttatgcatttatgcctacagc -3’ | 1915^a^ |
| Mb1R | Antisense | 5’-tggtggagttccactgcatagttaagtgggggtgagt -3’ | 118^a^ |
| Mb2F | Sense | 5’- actcacccccacttaactatgcagtggaactccacca -3’ | 3207^b^ |
| A1-F | Sense | 5'-gg**ggtacc**acatgttaagaaacttt-3' | 1050^a^ |
| ΔP-R | Antisense | 5'-gaatgggtgc***g***ttaggag-3' | 2438^a^ |
| ΔP-F | Sense | 5'-ctcctaa***c***gcacccattc-3' | 2421^a^ |
| A2-R | Antisense | 5'-aaaactgcaggccaccatgctgctat-3' | 3052^a^ |

The bold letters indicated the restriction site of *Hpa*I (gttaac) and *Kpn*I(ggtacc), respectively.

*: The bases (nt 967-972) were mutated to create the restriction site of *Hpa*I (gtcaat gttaac), without changing the deduced amino acid sequence.

^a^ The numbering of the WHV genome is according to the Genbank accession no. J04514.

^b^ The numbering of the HBV genome is according to the Genbank accession no. AY220698.
