# Supplementary material for: Persistence of the Recombinant Genomes of Woodchuck Hepatitis Virus in the Mouse Model
Source: PLoS One. 2015 May 5;10(5):e0125658. doi: 10.1371/journal.pone.0125658 (PMC4420481; doi:10.1371/journal.pone.0125658)
Supplement: S3 Table — (DOC) [file pone.0125658.s010.doc]

**S3 Table. Detection of viral DNA in sera, serum HBsAg, and hepatic WHcAg expression in mice received HI with pWHV-HBV-Sa, pWHV-HBV-SS, and pWHV-HBV-MS at week 45.**

| **Mice received HI with pWHV-HBV-Sa** | | | | | | | |
| --- | --- | --- | --- | --- | --- | --- | --- |
|  | **Sa1** | **Sa2** | **Sa3** | **Sa4** | **Sa5** | **Sa6** |  |
| **Viral DNA in sera** | Neg. | Neg. | Neg. | Pos. | Neg. | Neg. |  |
| **Serum HBsAg** | 1.235 | 0.001 | 1.156 | 1.45 | 0.898 | 0.009 |  |
| **Hepatic WHcAg expression** | Pos. | Neg. | Pos. | Pos. | Pos. | Neg. |  |
| **Mice received HI with pWHV-HBV-SS** | | | | | | | |
|  | **SS1** | **SS2** | **SS3** | **SS4** | **SS5** | **SS6** | **SS7** |
| **Viral DNA in sera** | Neg. | Neg. | Neg. | Neg. | Pos. | Pos. | Neg. |
| **Serum HBsAg** | 0.005 | 0.006 | 0.004 | 0.006 | 0.003 | 0.007 | 0.008 |
| **Hepatic WHcAg expression** | Neg. | Neg. | Neg. | Neg. | Neg. | Neg. | Neg. |
| **Mice received HI with pWHV-HBV-MS** | | | | | | | |
|  | **MS1** | **MS2** | **MS3** | **MS4** | **MS5** | **MS6** | **MS7** |
| **Viral DNA in sera** | Neg. | Neg. | Neg. | Neg. | Neg. | Neg. | Neg. |
| **Serum HBsAg** | 0.003 | 0.002 | 0.003 | 2.93 | 2.79 | 0.005 | 0.005 |
| **Hepatic WHcAg expression** | Neg. | Neg. | Neg. | Pos. | Pos. | Neg. | Neg. |

Neg.: negative; Pos.: positive. The results of HBsAg detection by ELISA are given as OD450 values with 0.1 as the cut off value.
